# The Tonsils, (Faucial, Lingual, Pharyngeal and Discrete); Their Functions and Relation to Affections of the Throat and Nose

**Published:** 1889-01

**Authors:** Scanes Spicer


AMERICAN JOURNAL

—OF—

DENTAL SCIENCE.

VOL. XXII. THIRD SERIES—JANUARY, 1889. NO. 9.

ARTICLE I.

### THE TONSILS (FAUCIAL, LINGUAL, PHARYNGEAL AND DISCRETE); THEIR FUNCTIONS AND RELATION TO AFFECTIONS OF THE THROAT AND NOSE.

BY SCANES SPICER, M. D. LOND.,

[Physician to the Throat Department of St. Mary's Hospital.]

It has long been known that lymphoid tissue, especially in the form of lymph follicles, is very extensively massed together in the naso-pharynx and pharynx. The most conspicuous of these masses, the faucial tonsils, are very familiar pathologically. Likewise is that mass known as the pharyngeal or Luschka's tonsil, situated in the vault of the naso-pharynx, and which, when hypertrophied, forms post-nasal growths. To a less extent is appreciated that aggregation of crypts and follicles called the lingual or fourth tonsil, situated at the base of the tongue between the circumvallate papillæ aud the epiglottis, although its pathological conditions are among the most fruitful causes of many of the paræsthesiæ and dysæsthesiæ of the throat. To the above must be added those discrete patches of follicles on the pharyngeal walls which form the anatomical lesion in chronic granular pharyngitis, and an enumeration has been made of the principal groups concerned in morbid states of this area. To understand why these various tonsils are so pre-eminently and conspicuously affected in diseases of the throat and nose, it is necessary to glance at their relations to adjacent structures and to the functions which they perform in the channels in which they are found, as far as is known; for there can be no doubt that such very large masses, exhibiting, moreover, some complexity of arrangement and sharing so readily in disease, must serve in the performance of some very important physiological function or functions.

*The Faucial Tonsils.*—Taking first the faucial tonsils, it must be remarked that physiologists have been very much in the dark as to their true functions, if we are to judge by the statements, or absence of statements, in the different English text-books of physiology; and physicians and laryngologists have not elucidated the matter much. The prevailing view appears to be that the faucial tonsils are essentially organs for the secretion of a lubricating fluid to aid in moistening the bolus of food before deglutition. Now, as the faucial tonsils are developmentally portions of the mucous membrane, there is no reason why they should not be provided with mucous glands to the same extent as the rest of the mucosa, and so secrete a little lubricating fluid, though insignificant in amount compared with that from the salivary glands. Bnt that this secretion is a quite subsidiary and unimportant function of the faucial tonsils is palpable from the facts that in any tonsil, healthy or diseased, infantile or adult, the component tissue is lymphoid, arranged in follicles, with more or less fibrous tissue, and that the secreting mucous glands are very scanty, and, indeed, in the ordinary excised tonsil not demonstrable. No doubt, when these organs have been excised for hypertrophy, the fibroid changes have caused atrophy of whatever gland tubes there might have been. Such fanciful theories as that the faucial tonsils are developed as compensatory organs for warming the inspired air when there is nasal obstruction, that they are reservoirs of nutriment like adipose tissue, and that their function is to keep the liquor amnii from passing into the fœtal pharynx, require no refutation here. It is to Dr. Hingston Fox in his admirable paper * that we are chiefly indebted for a lucid exposition as to some of the main functions of the faucial tonsils. His conclusions are, firstly, that the faucial tonsils act in the prevention of fluid waste in the economy by reabsorbing the buccal secretions to a large extent after their work is done, and especially in the intervals of the deglution acts; secondly, that they absorb certain of the elements of the food bolus as it is squeezed past them in deglutition; and, thirdly, that they form part of the blood-manufacturing system, and use up any nutriment remaining in the spent buccal secretions, acting, as he poetically expresses it, as "nurseries for young leucocytes, planted by the waterside, and drawing their sustenance from the nutrient stream." The facts on which these views are based may be classified as follows:—Firstly, the *anatomical*. The tonsils are like sponge in texture, consistence, and structure, being riddled with lacunæ or crypts. Every bolus of food must be squeezed against them as it is swallowed, a condition most favourable for the transfer of soluble matters. Then, in the intervals of deglutition, these spongy organs lie in the glosso-epiglottic fossæ, soaking in the buccal secretions, which fill up all their pores, and are delayed in their passage to the pharynx, if not entirely absorbed. Further, the tonsils are constructed on the type of a mucous membrane corrugated so as to expose a large surface to something, and on these corrugations are thickly-studded lymph follicles, as well as in these organs a very rich plexus of

---

* *The Lancet* and "Journal of Anatomy and Physiology," 1886

lymphatic vessels, which must have some function, and what more probable than the relation suggested of which we have so much confirmatory evidence? Also, these follicle aggregations are situated at places just below the output of the buccal secretions, and in the course which these must take. Secondly, the *histological.* The faucial tonsils are composed of tissue—lymphoid follicles—almost identical with that of the essentical parts of the blood-manufacturing system, the spleen, and lymphatic glands. Moreover, these adenoid follicles are densely crowed with leucocytes in all stages of development and with divided nuclei. The lymphatic vessel plexus throughout the tonsils is one of the richest in the body. Thirdly, the *physiological.* The faucial tonsils have very free arterial blood supply, which implies very considerable work done. Next it is a general law that fluids thrown out in the intestinal canal are absorbed by the segment of the intestine below, and in this area we have similar structures, the solitary and agminated glands, which are not found elsewhere in the body. Then, as a general rule, in health the tonsils atrophy in middle and late life, when blood manufacture is less active, and, on the other hand, tend to be large in children, when lymphoid tissues elsewhere are abundant and active, and blood manufacture is at its climax for the rapid processes of growth and nutrition. The considerations already stated render the correctness of the above views most probable, and it is the object of this paper to show that clinical and pathological facts harmonise with and corroborate them; and also support the view that other tonsils are largely channels of absorption.

*The Pharyngeal Tonsil.*—The anatomy and histology of this body in all essentials is that of the faucial tonsils; there are not so many crypts, nor are they so deep or subdivided, but there are differences of degree. These facts alone would tend to show that its function is not dissimilar—viz., the prevention of waste of some secretion. Now, in the horizontal position of the body, in man, all the nasal and lacrymal secretions are bound to flow over it. Here, then are all the conditions required on the hypothesis above enunciated; and when the facts from disease are added, the conclusion will be unavoidable that this tonsil saves and elaborates the spent nasal and lacrymal secretions.

*The Lingual Tonsil.*—An obseivation was recorded many years ago by Dr. Horace Dobell in *The Lancet*, and reprinted in his work on "Winter Cough" (third edition), that the uvula serves to convey the nasal secretions on to the base of the tongue in a plane anterior to the epiglottis, so keeping the constantly dripping fluids out of the larynx. This view was independently arrived at by me, and published in *The Lancet* and in the *British Medical Journal* (vol. i., 1888); but having since found that I have been anticipated by Dr. Dobell, I taking this opportunity of crediting him with priority. My paper, however, read before the Harveian Society in 1888, also pointed out, that in the erect posture these nasal and lacrymal secretions were dripped by the uvula on to an aggregation of crypts and follicles on the base of the tongue, such as was concerned elsewhere in the reabsorption of fluids and blood manufacture, and claimed that the same functions took place here. It is obvious that the buccal secretions also, to some extent, come in contact with the lingual tonsil; also that any portions of fluid not dealt with by the tonsils find the epiglottis keeps them out of the larynx, and run along the grooved lateral spouts of the epiglottis into the pyriform sinuses or hyoid fossæ, when they are swallowed.

Before considering pathological causes which affect pre-eminently one or the other tonsil, it might be as well to state here that usually all the tonsils are more or less involved, and that anatomical lesions are rarely confined to any one.

Commencing first with the pharyngeal tonsil, it must be premised that its morbid conditions are connected with the respiratory current and the channel in which it lies. Of all proximate causes of affections of this body, the most frequent is chronic nasal catarrh, and I shall therefore offer no apology for suggesting an explanation of its genesis. I believe it may be sought in the extreme variations in the temperature, humidity, and purity of the air breathed by civilised house-dwelling mankind, and, consequently, the great variation in the amount of moistening, warming, and filtering that has to be performed by the erectile mechanism of the nose. The savage, living in a state of nature, does not many times a day rapidly change his air currents from a temperature of 80° or more, and often laden with organic impurity and scorched up by a stove, for one frequently near the freezing point, and of widely different degrees of humidity and purity. The air he breathes is of a fairly constant quality comparatively by day and night. Hence there is a certain normal accommodation of his nasal erectile tissues to the work they have to do, which is not suddenly, frequently, or very materially departed from. We have in these considerations a possible explanation of the freedom of the Red Indians from catarrh, and the effectiveness of their nasal channels for breathing purposes as described by Catlin, who spent many years among them. On the other hand, with us civilised moderns, the frequent and sudden changes lead to corresponding activity in the erectile mechanism of the nose, and this repeated for months and years causes the erectile tissues to get into a state of irritable weakness, and to be permanently erected. There is then chronic congestion and discharge of a secretion differing somewhat from the normal. The exciting causes being constantly in action, chronic rhinitis is produced, then hypertrophy of the mucosa and narrowing of the passage. All this time the more or less perverted and acrid secretions have replaced the healthy ones, and have passed back constantly to the pharyngeal tonsil, which, having to tackle irritating secretions, gets swollen, inflamed, and in time hypertrophied, forming post-nasal growths, with all their attendant evils.

Having had constantly under my observation some hundreds of children for the last five years, I have been able over and over again to trace the whole development of post-nasal growths from ordinary chronic nasal catarrh, and that in children who have lived under the most favorable hygienic *regime*, except in as far as the above mentioned variations in the physical characters of the inspired air go; and it is to these variations alone that I can attribute the sequence of events. This state of chronic inflammation and debility of the tissues of the upper respiratory tract is not distinguishable from struma, and is often associated with general anæmia and debility, lympathic gland affection of the neck, concomitant affections of the conjunctivæ and ear (probably extension of inflammatory mischief up the nasal ducts and Eustachian tubes), and also by the forced substitution of buccal for nasal respiration, leading to depressed vitality of the tissues of the rest of the respiratory tract, facilitating the supervention of pneumonia and bronchitis, and preparing the soil for the invasion of the tubercle bacillæ. The above account indicates correctly, I believe, the relation between chronic nasal catarrh and pharyngeal tonsil hypertrophy, and also between the latter and the other morbid states referred to. But there are other causes than this, both of nasal catarrh and of acute inflammation of pharyngeal tonsil, the chief one among them being mechanical irritants, such as ordinary dust, trade dust, pollen, or other finely divided matter, which, carried in by the respiratory current, ultimately find their way back to the pharyngeal tonsil in the secretions which flush the passages, thus causing direct irritation by their presence. In the same way the germs of the specific fevers —measles, scarlet fever, variola, &c.—reach the pharyngeal tonsil and cause it to inflame, block the nasal passages in varying degrees, and, by damming back secretions, to produce anterior rhinorrhœa; similarly Eustachian obstruction, retention of secretions, ear abscesses, otorrhœa, and life-long deafness. Attention to the nose and naso-pharynx in specific fevers will be likely to prevent the conditions which are not susceptible of cure. Another cause ot pharyngeal tonsil enlargement is to be found in its absorption of irritant matters regurgitated or vomited by infants into their naso-pharynx. This often produces an acute coryza and nasal obstruction, independent of mechanical occlusion of nares by vomited matter, and due to direct irritation and enlargement of pharyngeal tonsil. Irritating vaginal secretions introduced during parturition and exciting snuffles is a well-known cause of posterior nasal obstruction from inflamed pharyngeal tonsil. It is a striking clinical fact that there is an overwhelming preponderance of pharyngeal tonsil mischief in the young. Is the cause to be sought in the smallness of the naso-pharynx, the early blocking of the nose, and the consequent stagnation and decomposition of the retained secretions? Judging from the anterior rhinorrhœa of such an irritating character as to cause eczema and the foulness of the secretions dislodged in the digital examination of post-nasal growths, I conclude that this is often so, and that the decomposing secretions keep up the enlargement commenced by an ordinary catarrh.

The lingual tonsil next requires consideration, as it is in relation with the same secretions as the pharyngeal, only especially in the erect position. All that has been said, therefore, as to the causes of chronic catarrh of the upper respiratory tract applies with equal force to lingual as to pharnygeal tonsil diseases, and in this conveyance of the acrid products of inflammation we have the main cause of its abnormal states. But there are many causes besides, especially the presence of deleterious and irritatıng matters in the alimentary ingesta. I would especially mention alcohol, condiments, very hot fluids, very cold fluids, or frequent alterations of these. Each of these can at times be distinctly traced as the exciting cause of lingual tonsil hypertrophy, which, in its turn, is the anatomical fact in the productions of the most obstinate and otherwise incomprehensible paræsthesiæ. In the case of this tonsil, too morbid influences derived from vitiated blood and secretions are very manifest. I refer especially to gout and rheumatism. Both the nasal and buccal secretions are surcharged with the poisons of those diseases periodically, and there can be no doubt that the inflammatory condition of lingual tonsil seen, and central angina and constriction complained of, so often in these diseases, and which often precede other symptoms or are alone present, are due to the irritation of the lingual tonsil by the perverted secretions. Evidence confirmatory of this view is found in the fact that anti diathetic treatment speedily cures the condition. The lingual tonsil does not show the same predisposition to be affected in syphilis as do the faucial tonsils. I venture to suggest that the syphilitic poison is excreted with the buccal secretions rather than with the nasal; and, as has already been stated, it is with the former that the faucial tonsils are in special correlation. An interesting observation bearing on lingual tonsil affection in scarlet fever was recorded to me by a pathologist who asked an explanation. In a fatal case he had observed ulcerations and erosions localised in the area of this organ. This pointed to the fact that the secretions loaded with germs and inflammatory products had vented their fury here, and seems singularly confirmatory of the importance of the lingual tonsil. Taking lupus of the nose and throat again, and infiltrations ulcerations have been found localized in the lingual tonsil area (Chiari and Reil), the infection having clearly been carried over healthy parts to a place where it could enter with the secretions and reproduce the disease. When there is nasal obstruction and substituted mouth-breathing, all the impurities of the environing atmosphere enter the mouth, and many of them alighting on the mucous membrane, are washed on to the lingual and faucial tonsils. Moreover, work is cast on the linings of the mouth—that of warming and moistening the air—which does not belong to them. Hence drying of the surface and failure of the secretions to wash away the decomposing *debris*. In the morning the patient complains of a dry mouth and a slight sore throat, due to inflammation of the lingual and faucial tonsils. This at first passes off during the day, but after some time leads to hypertrophy of the irritated structures. Other frequent and potent causes of lingual tonsil abnormalities are tobacco smoking, chewing, and snuffing, their action being irritant to the lymph follicles throughout the upper respiratory tract. Septic influences, bad teeth, neglected dirty teeth, false teeth not kept clean or not well fitting, must be added to the list of common causes of lymph follicle irritation and hypertrophy throughout the whole area under consideration, and must be attended to before a cure can be expected. Lingual tonsil mischief is specially met with in adults, and not so much in children. The reasons I would suggest are that the latter do not indulge in alcohol, condiments, or very hot or very cold fluids; and also that the irritating secretions which in children are penned up in the nose or directed forwards owing to enlarged pharyngeal tonsil to produce a rhinorrhœa, in adults pass on (there being more room in the naso-pharynx) to the lingual tonsil.

Concerning the faucial tonsils, the special relation of these to the buccal secretions has been referred to, and also that the factors in the production of disease of the other tonsils affect these likewise. It is unnecessary, therefore, to recapitulate, but it remains for me to remark additional facts. Very often unilateral hypertrophy of a faucial tonsil is seen. How often is it not in relation with a carious tooth on the same side, constantly contaminating the buccal secretions on that side? There can be little doubt that the tonsils are the sites where the poison of scarlatina, measles, and diphtheria usually enter the system, since they are the parts first and most constantly, and often alone, visibly affected; and the lymphatic glands in direct communication with them most markedly, soonest, and most frequently involved. Faulty voice production and excessive use of the voice are very important causes of affections of all the tonsils. Excessive use demands excessive lubrication, and the latter implies excessive absorption on the part of all the lymphoid follicles. Hence the diffusiveness, chronicity, and obstinacy of the changes met with in the throats of clergymen, actors, and Board School teachers. Regarding lacunar or so-called follicular tonsillitis (which, by the way, I have noticed more than once well marked in the crypts of the lingual tonsils), there are two distant varieties clinically. The first is that in which superficial inflammation and swelling of the mucosa blocks the crypt orifices and causes retention of the shed epithelium and *debris*. This form is acute and usually easily curable, and may be brought about by septic or common catarrhal causes. It is painful, and requires sedative and antiphlogistic treatment. This variety often forms the starting-point of a lacunar abscess, or the process may extend deeper and peri-tonsillar abscess or quinsy ensue. The second form is chronic, and depends on a natural or acquired sluggishness with which the desquamation processes of the epithelium lining the tonsillar crypts are performed, and the *debris* is not normally extruded by a *vis a tergo*. This is not septic or painful, though there may be slight stiffness and discomfort. It is very chronic in course, and difficult to overcome, yielding best, however, to solvent and stimulant local applications. The discrete nodules in the pharyngeal walls partake in the morbid processes going on in the other tonsils, and are similarly affected by the causes acting on them.

It now remains for me to state that it has not been my object in this paper to give details of symptoms and treatment, and merely to indicate the lines upon which nose and throat affections should be treated when, as is generally the case, the various tonsils—the points of maximum irritation—show palpable signs of morbid action; and it must be observed that, when the various methods of treatment in use in the past are considered in the light of the above views as to the functions of the tonsils, it will be seen that these views afford a scientific explanation of the success of those empirical methods which have been hitherto the most approved. These lines would be, firstly, the ensurance of physiological rest to the affected tissues by arresting morbid and lessening profuse secretions, and promoting derivative action into other channels. In acute and sub-acute affections, a blue pill, followed by regular small doses of belladonna, gives excellent results. Secondly the removal of all causes of irritation and inflammation in the inspired air, whether due to occupation, habits, or conditions of existence, and a similar regulation of the habitual alimentary ingesta. Thirdly, the soothing of any acute inflammation or pain by ordinary measures, ice, bland fluids, jelly, cocaine, &c. Fourthly, the attack of any diathetic condition which may be causing perversion of secretions—as gout, rheumatism, syphillis, &c.,—on general principles. Fifthly, the removal of any hypertrophied tissue—such as enlarged tonsils, post-nasal growths, hypertrophied lingual tonsil, granules of granular pharynx, &c.,—which may be occluding any physiological channel or causing mechanical irritation of adjacent parts by the numerous approved methods at our disposal. Sixthly, the prevention of the accumulation or stagnation of any of the secretions of the nose, mouth, or pharnyx by cleaning and antiseptic washes.

In conclusion, I would say—1. The significance of the various tonsils is in their palpable relation to the blood-manufacturing system and to the outpour of copious secretions. The relations of the tonsils to the rest of the organism can be well appreciated by comparing them with the relations of the sewage farm to the town whose refuse it makes use of, and to which it returns its elaborated products. 2. If any of the secretions delivered to the tonsils become contaminated in any way with irritating matter, whether generated in the system or introduced from without, those tonsils in physiological correlation with the affected secretion show irritative changes varying in degree.

The function and affections of the various tonsils afford the key to the comprehension and scientific treatment—and the prevention—of many of the most intractable and recurrent disorders of the nose and throat.—*London Dental Record.*

---

ARTICLE II.

### IMMEDIATE FILLING OF ROOT CANALS.

